# Acute activation of hemichannels by ethanol leads to Ca^2+^-dependent gliotransmitter release in astrocytes

**DOI:** 10.3389/fcell.2024.1422978

**Published:** 2024-06-21

**Authors:** Gonzalo I. Gómez, Claudia García-Rodríguez, Jesús E. Marillán, Sergio A. Vergara, Tanhia F. Alvear, Arantza Farias-Pasten, Juan C. Sáez, Mauricio A. Retamal, Maximiliano Rovegno, Fernando C. Ortiz, Juan A. Orellana

**Affiliations:** ^1^ Faculty of Health Sciences, Institute of Biomedical Sciences, Universidad Autónoma de Chile, Santiago, Chile; ^2^ Centro Interdisciplinario de Neurociencia de Valparaíso, Facultad de Ciencias, Instituto de Neurociencia, Universidad de Valparaíso, Valparaíso, Chile; ^3^ Departamento de Neurología, Escuela de Medicina and Centro Interdisciplinario de Neurociencias, Facultad de Medicina, Pontificia Universidad Católica de Chile, Santiago, Chile; ^4^ Programa de Comunicación Celular en Cancer, Facultad de Medicina Clínica Alemana, Universidad del Desarrollo, Santiago, Chile; ^5^ Departamento de Medicina Intensiva, Facultad de Medicina, Pontificia Universidad Católica de Chile, Santiago, Chile; ^6^ Mechanisms of Myelin Formation and Repair Laboratory, Departamento de Biología, Facultad de Química y Biología, Universidad de Santiago de Chile, Santiago, Chile

**Keywords:** alcoholism, connexins, pannexins, glia, hemichannels, connexin 43, pannexin-1

## Abstract

Multiple studies have demonstrated that acute ethanol consumption alters brain function and cognition. Nevertheless, the mechanisms underlying this phenomenon remain poorly understood. Astrocyte-mediated gliotransmission is crucial for hippocampal plasticity, and recently, the opening of hemichannels has been found to play a relevant role in this process. Hemichannels are plasma membrane channels composed of six connexins or seven pannexins, respectively, that oligomerize around a central pore. They serve as ionic and molecular exchange conduits between the cytoplasm and extracellular milieu, allowing the release of various paracrine substances, such as ATP, D-serine, and glutamate, and the entry of ions and other substances, such as Ca^2+^ and glucose. The persistent and exacerbated opening of hemichannels has been associated with the pathogenesis and progression of several brain diseases for at least three mechanisms. The uncontrolled activity of these channels could favor the collapse of ionic gradients and osmotic balance, the release of toxic levels of ATP or glutamate, cell swelling and plasma membrane breakdown and intracellular Ca^2+^ overload. Here, we evaluated whether acute ethanol exposure affects the activity of astrocyte hemichannels and the possible repercussions of this phenomenon on cytoplasmatic Ca^2+^ signaling and gliotransmitter release. Acute ethanol exposure triggered the rapid activation of connexin43 and pannexin1 hemichannels in astrocytes, as measured by time-lapse recordings of ethidium uptake. This heightened activity derived from a rapid rise in [Ca^2+^]_i_ linked to extracellular Ca^2+^ influx and IP_3_-evoked Ca^2+^ release from intracellular Ca^2+^ stores. Relevantly, the acute ethanol-induced activation of hemichannels contributed to a persistent secondary increase in [Ca^2+^]_i_. The [Ca^2+^]_i_-dependent activation of hemichannels elicited by ethanol caused the increased release of ATP and glutamate in astroglial cultures and brain slices. Our findings offer fresh perspectives on the potential mechanisms behind acute alcohol-induced brain abnormalities and propose targeting connexin43 and pannexin1 hemichannels in astrocytes as a promising avenue to prevent deleterious consequences of alcohol consumption.

## 1 Introduction

Acute alcohol intoxication has far-reaching effects on the central nervous system (CNS), leading to compromised decision-making (George et al., 2005), heightened aggression (Massa et al., 2019), reduced ability to intervene in bystander situations (Leone and Parrott, 2019), and a higher propensity for intoxicated driving despite the risks (Motschman et al., 2020). The effects of acute alcohol intoxication begin with mild impairments in tasks requiring skills, along with dysmetria, slower reaction times, increased talkativeness, and relaxation at blood alcohol levels (BAL) of 5–20 mM (Jung and Namkoong, 2014). At higher BAL (20–40 mM), individuals experience slowed thinking and altered cognitive processing. When the BAL reaches 40 mM and above, some may even encounter memory “blackouts,” where they engage in complex behaviors but have no memory of them afterward (White, 2003). These blackouts indicate a temporary inability to form new memories and can lead to severe outcomes. When BAL exceeds 86.8 mM, severe consequences like respiratory depression, coma, and potentially death can occur (Vonghia et al., 2008).

Early onset of alcohol intoxication is a significant risk factor for later alcohol bingeing and the development of alcohol addiction (DeWit et al., 2000; Morean et al., 2014; Whelan et al., 2014), a chronically relapsing condition characterized by compulsive alcohol-seeking and consumption (Carvalho et al., 2019). While many studies have focused on the effects of chronic alcohol exposure on the brain, our understanding of the neural adaptations triggered by acute alcohol intake remains incomplete. It is crucial to understand how ethanol directly affects brain networks to pinpoint new therapeutic targets for reducing excessive drinking and mitigating the public health consequences of acute alcohol intoxication. In recent years, an emerging idea suggests that ethanol-induced brain changes may partly be influenced by its direct effects on non-neuronal cells, particularly astrocytes (Hong et al., 2021; Kim et al., 2022; Coulter et al., 2023). Among other key functions, such as energy supplier or extracellular K^+^ buffering, astrocytes play a crucial role in the “tripartite synapse,” which is central to chemical synaptic transmission (Allen and Eroglu, 2017). They sense neural activity and respond by releasing bioactive molecules called “gliotransmitters,” a process tightly dependent on intracellular free Ca^2+^ concentration ([Ca^2+^]_i_) (Araque et al., 2014). These gliotransmitters regulate cerebral blood flow, facilitate energy-rich metabolite exchange, and contribute to immune response and brain interstitial fluid homeostasis (Weber and Barros, 2015; Verkhratsky and Nedergaard, 2018). Ethanol, the main component of alcoholic beverages, triggers acute effects on astrocytes, including increased expression of NF-κB, iNOS and COX-2 (Blanco et al., 2004; Blanco et al., 2005). Furthermore, ethanol causes a rapid increase in [Ca^2+^]_i_ and disrupts the communication between astrocytes, as well as the release of gliotransmitters (Kimelberg et al., 1993; Allansson et al., 2001; Adermark et al., 2004; Kim et al., 2022). However, the mechanisms behind these alterations and the profound impact of astroglial dysfunction on ethanol-induced brain abnormalities are still unclear.

Recently, our group conducted a couple of studies that have highlighted the activation of hemichannels as a novel mechanism through which long-lasting exposure to ethanol disrupts astrocyte state (Gómez et al., 2018; Gómez et al., 2024). This involves a cascade of inflammatory pathways being sequentially stimulated, ultimately resulting in astrocyte damage. Unlike most plasma membrane channels, which selectively allow ions such as K^+^, Na^+^, and Cl^−^ to pass through, hemichannels serve as conduits for both ions and small molecules due to their larger pore diameters (Montero and Orellana, 2015). Hemichannels are made up of six connexin or seven pannexin monomers surrounding a central pore, facilitating the passage of substances between the cytosol and the extracellular space (Giaume et al., 2013). While connexin and pannexin hemichannels both fall within the category of large-pore channels (Syrjanen et al., 2021), they differ significantly in terms of amino acid sequence, permeability, conductance, as well as gating and posttranslational mechanisms that regulate them (D'Hondt et al., 2013; Patel et al., 2014; Esseltine and Laird, 2016). In the CNS, astrocytes express functional hemichannels composed mainly by connexin 43 (C× 43) and pannexin-1 (Panx1), facilitating the release of gliotransmitters essential for synaptic transmission, plasticity, behavior, and memory (Chever et al., 2014; Meunier et al., 2017; Cheung et al., 2022; Linsambarth et al., 2022; Vasile et al., 2022). However, during pathological conditions, the heightened activity of these channels in astrocytes contributes to homeostatic disturbances associated with the pathogenesis and progression of various brain diseases (Garré et al., 2016; Chávez et al., 2019; Gajardo-Gómez et al., 2020; Almad et al., 2022; Guo et al., 2022). Research suggests that uncontrolled influx of Na^+^ and Cl^−^ through hemichannels can lead to osmotic and ionic imbalances, resulting in aquaporin-mediated cell swelling and plasma membrane breakdown (Paul et al., 1991; Diaz et al., 2019). Moreover, the persistent and dysregulated opening of hemichannels, which are permeable to Ca^2+^ and/or indirectly increase [Ca^2+^]_i_, may lead to [Ca^2+^]_i_ overload, triggering the production of free radicals, lipid peroxidation, and plasma membrane damage (Giaume et al., 2021). Alternatively, exacerbated activity of these channels could also induce the release of potentially harmful molecules such as glutamate, ATP, and D-serine to neighboring cells (Orellana et al., 2016).

In this study, we demonstrate that acute ethanol exposure rapidly increases the activity of hemichannels in cortical astrocytes and in HeLa cells expressing either Cx43 or Panx1. The latter response depended on a rise in [Ca^2+^]_i_ driven by extracellular Ca^2+^ influx and IP_3_-induced Ca^2+^ release from intracellular stores. Importantly, the acute ethanol-induced activity of astroglial hemichannels resulted in increased release of ATP and glutamate, as observed in culture and brain slice preparations.

## 2 Material and methods

### 2.1 Reagents and antibodies

HEPES, water (W3500), ethanol, DNAse I, poly-L-lysine, ATP, glutamate determination kit and probenecid (Prob) were purchased from Sigma-Aldrich (St. Louis, MO, United States). Fetal bovine serum (FBS) was obtained from Hyclone (Logan, UT, United States). Penicillin, streptomycin, Trypsin 10×, Hank’s solution, ATP determination kit, Dulbecco’s Modified Eagle Medium (DMEM), Phosphate-Buffered Saline (PBS) and ethidium (Etd) bromide (10 mg/mL) were purchased from Thermo Fisher Scientific (Waltham, MA, United States). Gap19 (KQIEIKKFK, intracellular loop domain of Cx43), gap19^I130A^ (KQAEIKKFK, negative control), TaT-L2 (YGRKKRRQRRRDGANVDMHLKQIEIKKFKYGIEEHGK, second intracellular loop domain of Cx43), TaT-L2^H126K/I130N^ (YGRKKRRQRRR-DGANVDMKLKQNEIKKFKYGIEEHGK, negative control), ^10^panx1 (WRQAAFVDSY, first extracellular loop domain of Panx1) and ^10^panx1 scramble (^10^panx1^scrb^, FSVYWAQADR) peptides were obtained from Genscript (New Jersey, United States).

### 2.2 Animals

Animal experimentation was conducted in accordance with the guidelines for the care and use of experimental animals of the US National Institutes of Health (NIH), the *ad hoc* committee of the Agencia Nacional de Investigación y Desarrollo (ANID), the Bioethics Committee of the Pontificia Universidad Católica de Chile (PUC) (n°: 200605010) and the guidelines of European Community Council Directives of 01/01/2013 (2010/63/EU) and the animal care committee of the Center for Interdisciplinary Research in Biology in College de France. C57BL/6 (PUC) mice of 8–9 weeks of age were housed in cages in a temperature-controlled (24°C) and humidity-controlled vivarium under a 12 h light/dark cycle (lights on 8:00 a.m.), with *ad libitum* access to food and water. Some experiments were carried out using mice of wild-type C57BL/6j background, mice expressing enhanced green fluorescent protein under the astrocytic promoter glial fibrillary acidic protein (GFAP-eGFP) provided by F. Kirchhoff (University of Saarland, Germany).

### 2.3 Cell cultures

Astroglial cell primary cultures were prepared from the cortex of postnatal day 2 (P2) mice as previously described (Avendano et al., 2015). Briefly, brains were removed, and cortices were dissected. Meninges were carefully peeled off and tissue was mechanically dissociated in Ca^2+^ and Mg^2+^ free Hank’s balanced salt solution with 0.25% trypsin and 1% DNAse. Cells were seeded onto 35-mm plastic dishes (Corning, NY, United States) or onto glass coverslips (Fisher Scientific, Waltham, MA, United States) placed inside 24-well plastic plates (Corning, NY, United States) at the density of 3 × 10^5^ cells/dish or 1x10^5^ cells/well, respectively, in DMEM, supplemented with penicillin (5 U/mL), streptomycin (5 μg/mL), and 10% FBS. Cells were grown at 37°C in a 5% CO_2_/95% air atmosphere at nearly 100% relative humidity. Following 8–10 days *in vitro* (DIV), 1 µM AraC was added for 3 days to suppress the proliferation of microglia. Medium was changed twice a week and cultures were used after 3 weeks. Parental HeLa cells knock-out for Cx45 (HeLa-KO45) were used to ensure no endogenous expression of this connexin. HeLa cells were stably transfected with mouse Cx43^EGFP^ or Panx1^EGFP^, with EGFP fused to the C-terminus of these proteins, as previously described (Salas et al., 2015; Harcha et al., 2019). Cells were cultured in low glucose DMEM media supplemented with 10% FBS as well as 50 U/mL penicillin and streptomycin at 37°C in a 5% CO_2_/95% air atmosphere. Cells were selected by their resistance to the antibiotic geneticin (G418, maintained with 1 mg/mL in the medium). The culture medium was replaced every other day.

### 2.4 Acute brain slices

Mice were anesthetized under isoflurane, decapitated and brains were extracted and cut into coronal slices (300 µm) using a vibratome (Leica, VT1000GS; Leica, Wetzlar, Germany) filled with ice-cold slicing solution containing (in mM): sucrose (222); KCl (2.6); NaHCO_3_ (27); NaHPO_4_ (1.5); glucose (10); MgSO_4_ (7); CaCl_2_ (0.5) and ascorbate (0.1), bubbled with 95% O_2_/5% CO_2,_ pH 7.4. The substitution of NaCl by sucrose reduces the Na^+^ driving force, minimizing Na^+^ entry into cells and thereby reducing excitotoxicity (Aghajanian and Rasmussen, 1989). Additionally, this slicing solution has a low Ca^2+^/high Mg^2+^ ratio, which ensures very low neural excitability. Then, the slices were transferred at room temperature (20°C–22°C) to a holding chamber in ice-cold artificial cerebral spinal fluid (aCSF) containing (in mM): NaCl (125), KCl (2.5), glucose (25), NaHCO_3_ (25), NaH_2_PO_4_ (1.25), CaCl_2_ (2), and MgCl_2_ (1), bubbled with 95% O_2_/5% CO_2_, pH 7.4, for a stabilization period of 60 min before dye uptake experiments (see below).

### 2.5 Treatments

Astrocytes or HeLa-cells were acutely treated with 0, 1, 10, 25, 50, or 100 mM of EtOH. The following pharmacological agents or conditions were used 30 min prior and during acute stimulation with ethanol during experiments: Ca^2+^-free solution, mimetic peptides against Cx43 (gap19: 50 μM; TaT-L2: 50 µM) and Panx1 (^10^panx1, 50 µM) hemichannels, mutated peptides against Cx43 hemichannels (gap 19^19I130A^: 50 μM; TaT-L2^H126K/I130N^: 50 µM), scramble peptide against Panx1 hemichannels (^10^panx1^scrb^, 50 µM), Probenecid (Prob, Panx1 hemichannel blocker, 200 µM), BAPTA-AM (intracellular Ca^2+^ chelator, 10 μM), thapsigargin (TG, compound that depletes the intracellular Ca^2+^ stores, 2 µM), xestospongin C (Xest-C, IP_3_ receptor blocker, 5 µM), xestospongin B (Xest-B, IP_3_ receptor blocker, 5 µM), ryanodine (general RyR receptor blocker, 100 μM) or dantrolene (RyR1 receptor blocker, 50 µM). Acute brain slices were acutely treated with 25 mM EtOH. Some acute brain slices were pre-incubated for 30 min before and during acute EtOH experiments with the following agents: gap19 (50 µM), ^10^panx1 (50 µM), BAPTA-AM (10 μM) or Xest-C (5 µM). In some experiments, hemichannel blockers were also used acutely to inhibit Etd uptake. Conditioned media (CM) from astrocytes or brain slices were obtained from supernatants collected after 10 min of acute ethanol exposure, filtered (0.22 µm), and stored at −80°C.

### 2.6 Dye uptake in cultured cells

For dye uptake experiments in astrocytes, they were plated on 12 mm glass coverslips and, after 2 weeks of culture, were washed twice in Hank´s balanced salt solution. Then, astrocytes were incubated at room temperature with recording solution (in mM): 148 NaCl, 5 KCl, 1.8 CaCl_2_, 1 MgCl_2_, 5 glucose, and 5 HEPES, pH 7.4, containing 5 μM Etd and mounted on the stage of an Olympus BX 51W1I upright microscope with a ×40 water immersion objective for time-lapse imaging. Images were captured by a Retiga 1300I fast-cooled monochromatic digital camera (12-bit) (Qimaging, Burnaby, BC, Canada) controlled by imaging software Metafluor software (Universal Imaging, Downingtown, PA) every 30 s (exposure time = 0.5 s; excitation and emission wavelengths were 528 nm 598 nm, respectively). For dye uptake experiments in HeLa cells, they were first seeded on 25 mm glass coverslips and used when they reached 70%–80% confluency. Then, they were bathed with recording Krebs solution (in mM): 118 NaCl, 4.7 KCl, 3 CaCl_2_, 1.2 MgCl_2_, 10 glucose, 20 HEPES, 9.9 Tris; pH 7.4; containing 5 µM DAPI. Fluorescence intensity images were recorded in cells that were selected for having a fluorescent label, indicating that they express Cx43^EGFP^ or Panx1^EGFP^. The images were taken with a NIKON Eclipse Ti inverted microscope (Japan) every 15 s for 5 min per condition. Nikon software (NIS Elements Advanced Research) was used for off-line image analysis. For astrocytes and HeLa cells, the fluorescence intensity recorded from at least 20 regions of interest (representing 20 cells per cultured coverslip) was calculated with the following formula: Corrected total cell fluorescence = Integrated Density—([Area of selected cell] x [Mean fluorescence of background readings]). The mean slope of the relationship over a given time interval (ΔF/ΔT) represents the dye uptake rate and was calculated with regression lines that were fitted to points before and after the various experimental conditions using Microsoft Excel (Seattle, WA, United States). The mean values of slopes were plotted using GraphPad Prism 7 software (La Jolla, California, United States) and expressed as AU/min. At least three replicates (four sister cultured coverslips) were measured in each independent experiment. In some experiments, cultured astrocytes or HeLa cells were pre-incubated with Cx43 and/or Panx1 channel blockers for 15 min before and during the time-lapse experiments of dye uptake: gap19 (50 µM), TaT-L2 (50 µM), ^10^panx1 (50 µM), gap19^I130A^ (50 µM), TaT-L2^H126K/I130N^ (50 µM), ^10^panx1^scrb^ (50 µM) or Prob (200 µM).

### 2.7 Intracellular Ca^2+^ imaging

Astrocytes plated on glass coverslips were loaded with 5 µM Fura-2-AM in DMEM without serum at 37°C for 45 min and then washed three times in Locke’s solution (154 mM NaCl, 5.4 mM KCl, 2.3 mM CaCl_2_, 5 mM HEPES, pH 7.4) followed by de-esterification at 37°C for 15 min. The experimental protocol for Ca^2+^ signal imaging involved data acquisition every 5 s (emission at 510 nm) at 340/380-nm excitation wavelengths, respectively, using an Olympus BX 51W1I upright microscope with a ×40 water immersion objective. Changes were monitored using an imaging system equipped with a Retga 1300I fast-cooled monochromatic digital camera (12-bit) (Qimaging, Burnaby, BC, Canada), monochromator for fluorophore excitation, and METAFLUOR software (Universal Imaging, Downingtown, PA) for image acquisition and analysis. Analysis involved determination of pixels assigned to each cell. The average pixel value allocated to each cell was obtained with excitation at each wavelength and corrected for background. Due to the low excitation intensity, no bleaching was observed even when cells were illuminated for a few minutes. The FURA-2 ratio was obtained after dividing the 340-nm by the 380-nm fluorescence image on a pixel-by-pixel base (R = F340 nm/F380 nm).

### 2.8 Dye uptake in acute brain slices

Time-lapse recordings of Etd uptake in brain slices were done in a submerged recording chamber perfused with oxygenated aCSF containing 20 μM Etd at a flow rate of 4–5 mL/min at room temperature (22°C). To stabilize the tissue and prevent it from moving within the fluid stream, we utilized a slice-anchor or “harp” constructed from nylon or gold threads stretched and secured across a U-shaped piece of gold or platinum wire. Image time series were acquired with a TCS SP5 upright two-photon microscope (Leica) with aHCX IRAPO, L 25x, NA = 0.95, water immersion objective (Leica). Fluorophores were excited at 800 nm using a Ti:Sapphire laser (MaiTai, −0.1 MW, Spectra-Physics, Santa Clara, CA, US). Laser power below the objective was kept between 20 and 40 mW to minimize laser-induced artefacts and phototoxicity. Fluorescence light was collected in the epifluorescence configuration. The Etd fluorescence was separated from the GFP fluorescence using a dichroic mirror (562 nm, Semrock, US). Fluorescence emissions were detected simultaneously by two non-descanned photomultiplier tubes with a 542/50 nm filter for “green” fluorescence emission and a 617/73 nm filter for “red” fluorescence emission. XY time-lapse series of basal and EtOH-induced Etd uptake were subsequently recorded for 30 min at 0.03 Hz, at a depth of 100–150 μm beneath the surface. Leica Application Suite Advanced Fluorescence (LASAF) and ImageJ/FIJI software were used for off-line image analysis. Etd fluorescence intensity was calculated with the following formula: Corrected total cell fluorescence = Integrated Density–([Area of selected cell] x [Mean fluorescence of background readings]). The mean slope of the relationship over a given time interval (ΔF/ΔT) represents the dye uptake rate and was calculated with regression lines that were fitted to points before and after the various experimental conditions using Microsoft Excel (Seattle, WA, United States). The mean values of slopes were plotted using GraphPad Prism 7 software (La Jolla, California, United States) and expressed as arbitrary units (AU)/min. In some time-lapse recordings, brain slices were acutely treated with the following inhibitors after acute treatment with EtOH: gap19 (50 µM) or ^10^panx1 (50 µM).

### 2.9 Measurement of extracellular ATP and glutamate concentration

Extracellular ATP in CM was measured using a luciferin/luciferase bioluminescence assay kit (Sigma-Aldrich), while extracellular levels of glutamate were determined using an enzyme-linked fluorometric assay (Sigma-Aldrich). Cells were lysed with a Tris-buffered solution containing 1% TritonX-100 and supernatants of whole-cell lysates were used for measurements of protein levels. The amounts of ATP and glutamate in the samples were calculated from standard curves and normalized for the protein concentration using the Bio-Rad protein assay.

### 2.10 Astrocyte morphology

Astrocytes plated on glass coverslips were incubated in Locke’s solution and then imaged using a Nikon Eclipse Ti2-E inverted microscope with a 60x, NA = 1.49, APO TIRF DIC oil immersion objective (Tokyo, Japan). Changes in differential interference contrast microscopy were monitored using an imaging system equipped with a Nikon DS-Fi2 Camera (Tokyo, Japan). XY time-lapse series of basal and acute EtOH exposure were subsequently recorded every 3 s for 5 min. ImageJ/FIJI software were used for off-line image analysis. Then, images were converted into binary (black-and-white) images through the default thresholding method (IsoData algorithm of ImageJ). The following parameters related to astroglial morphology were measured: “Area,” “Perimeter” and “Aspect ratio”. The mean values were plotted using GraphPad Prism 7 software (La Jolla, California, United States).

### 2.11 Data analysis and statistics

Detailed statistical results were included in the figure legends. Statistical analyses were performed using GraphPad Prism (version 7, GraphPad Software, La Jolla, CA). Normality and equal variances were assessed using the Shapiro-Wilk normality test and Brown-Forsythe test, respectively. Unless otherwise stated, data that passed these tests were analyzed by unpaired *t*-test in case of comparing two groups, whereas in case of multiple comparisons, data were analyzed by one or two-way analysis of variance (ANOVA) followed, in case of significance, by a Tukey’s *post hoc* test. A probability of *p* < 0.05 was considered statistically significant.

## 3 Results

### 3.1 Acute ethanol exposure increases the activity of Cx43 and Panx1 hemichannels in cultured astrocytes

**FIGURE 1:**
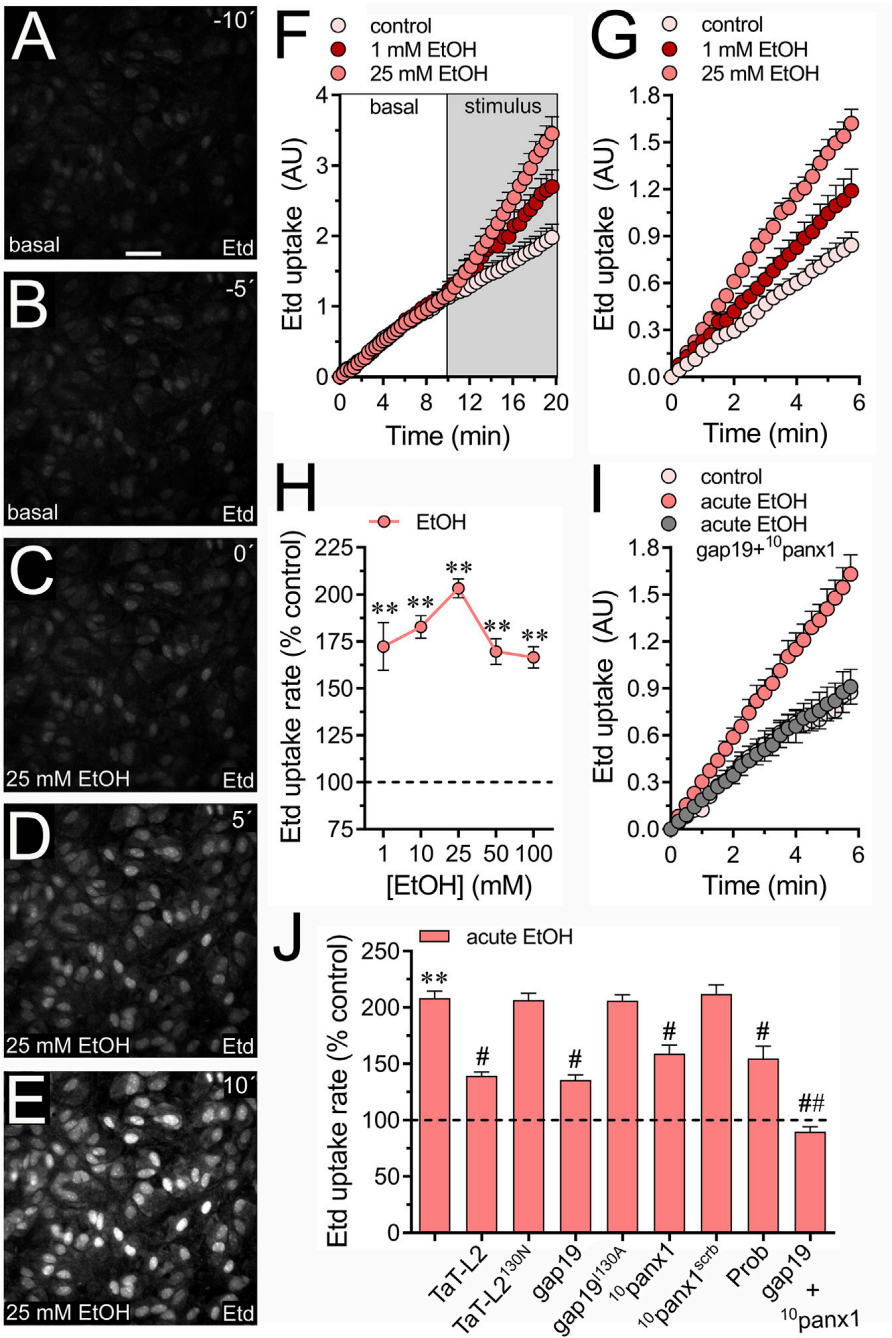
Acute ethanol exposure increases the activity of Cx43 and Panx1 hemichannels in cultured astrocytes. **(A–E)** Representative fluorescence micrographs illustrating time-lapse recordings of Etd uptake by astrocytes for 10 **(A)** and 5 **(B)** seconds before and after stimulation with 25 mM ethanol (EtOH) for 0 **(C)**, 5 **(D)**, or 10 **(E)** seconds. **(F)** Time-lapse measurements of Etd uptake by astrocytes under control conditions (pale pink circles) or after the acute treatment with 1 mM (dark red circles) or 25 mM (salmon red circles) ethanol. **(G)** Time-lapse measurements of Etd uptake by astrocytes under control conditions (pale pink circles) or treated for 1 min before and during recordings with 1 mM (dark red circles) or 25 mM (salmon red circles) ethanol. **(H)** Averaged Etd uptake rate normalized with the control condition (dashed line) by astrocytes acutely treated with different concentrations of ethanol (salmon red circles). **p < 0.005, ethanol treatment compared to control conditions (one-way ANOVA followed by Tukey’s *post hoc* test). **(I)** Time-lapse measurements of Etd uptake by astrocytes under control conditions (pale pink circles) or treated for 1 min before and during recordings with 25 mM ethanol alone (salmon red circles) or in combination with 50 µM gap19 + 50 µM ^10^panx1 (gray circles). **(J)** Averaged Etd uptake rate normalized with control condition (dashed line) by astrocytes acutely treated with 25 mM ethanol alone or in combination with the following blockers: 50 µM TaT-L2, 50 µM TaT-L2^H126K/I130N^, 50 µM gap19, 50 µM gap19^I130A^, 50 µM ^10^panx1, 50 µM ^10^panx1^scrb^, 500 µM Probenecid (Prob), and 50 µM gap19 + 50 µM ^10^panx1. **p < 0.0005, ethanol compared to control; ^#^ *p* < 0.05, ^##^ *p* < 0.005; effect of pharmacological agents compared to ethanol treatment (one-way ANOVA followed by Tukey’s *post hoc* test). Data were obtained from at least three independent experiments with three or more repeats each one (≥25 cells analyzed for each repeat). Calibration bar = 150 μm.

Acute stimulation with ethanol causes a rapid augment in the expression of NF-κB, iNOS and COX-2 (Blanco et al., 2004; Blanco et al., 2005) along with a rise in [Ca^2+^]_i_ and release of taurine and aspartate in astrocytes (Kimelberg et al., 1993; Allansson et al., 2001; Adermark et al., 2004; Kim et al., 2022). However, the molecular mechanisms underlying these alterations remain still unclear. We recently showed that long-lasting stimulation (hours to days) elevates the activity of Cx43 and Panx1 hemichannels in astrocytes (Gómez et al., 2024). Yet, it is unclear whether acute ethanol exposure (seconds to minutes) could affect the activity of these channels in primary cortical astrocytes. To evaluate the functional status of hemichannels in astrocytes, we measured the uptake rate of ethidium (Etd), a dye that enters the cytoplasm of healthy cells through plasma membrane channels with large pores (Johnson et al., 2016). Upon intercalating with base pairs of DNA and RNA, Etd becomes fluorescent, indicating channel activity. Time-lapse recordings showed that acute ethanol stimulation instantly doubled the Etd uptake rate in cultured astrocytes (Figures 1A–F). We observed similar responses when astrocytes were incubated with ethanol for 1 min and then placed on the microscope stage to record the Etd uptake rate over time (Figure 1G). The increase in Etd uptake induced by acute ethanol was concentration-dependent, peaking at 25 mM and gradually declining with higher concentrations (Figure 1H). Therefore, we decided to use 25 mM ethanol for our subsequent experiments.

Since both Cx43 and Panx1 hemichannels play a significant role in dye influx in astrocytes (Iglesias et al., 2009; Sáez et al., 2020), we investigated their potential involvement in the acute ethanol-induced Etd uptake. The contribution of Cx43 hemichannels to acute ethanol-mediated Etd uptake was explored using gap19 and TaT-L2, two mimetic peptides that inhibit Cx43 hemichannels by interacting with the intracellular L2 loop of Cx43 (Iyyathurai et al., 2013). Both gap19 (50 µM) or TaT-L2 (50 µM) effectively reduced the acute ethanol-induced Etd uptake in astrocytes, although not completely (Figures 1I,J). Furthermore, an inactive form of gap19, containing the I130A modification (gap19^I130A^), was ineffective in reducing the acute ethanol-induced Etd uptake in astrocytes (Figure 1J). Similarly, we observed that a modified TaT-L2 (TaT-L2^H126K/I130N^), in which two amino acids crucial for binding L2 to the CT tail of Cx43 are mutated, did not elicit a similar inhibitory response (Figure 1J). To examine the role of Panx1 hemichannels, we pharmacologically inhibited them with the mimetic peptide ^10^panx1 (50 µM) or probenecid (500 µM) (Pelegrin and Surprenant, 2006; Silverman et al., 2008). Both ^10^panx1 or probenecid partially attenuated the acute ethanol-induced Etd uptake in astrocytes (Figure 1J). Similar inhibitory responses were observed by employing the scrambled version of ^10^panx1 (^10^panx1^scrb^, 50 µM) (Figure 1J). Significantly, the simultaneous administration of gap19 and ^10^panx1 completely blocked acute ethanol-induced Etd uptake (Figure 1J), suggesting that acute ethanol exposure rapidly augments the activity of both Cx43 and Panx1 hemichannels in astrocytes. The acute ethanol-induced activation of hemichannels was not associated with changes in the area, perimeter, or aspect ratio of astrocytes (Supplementary Figure S1A). This indicates that astroglial morphology remained unchanged in our system.

**FIGURE 2:**
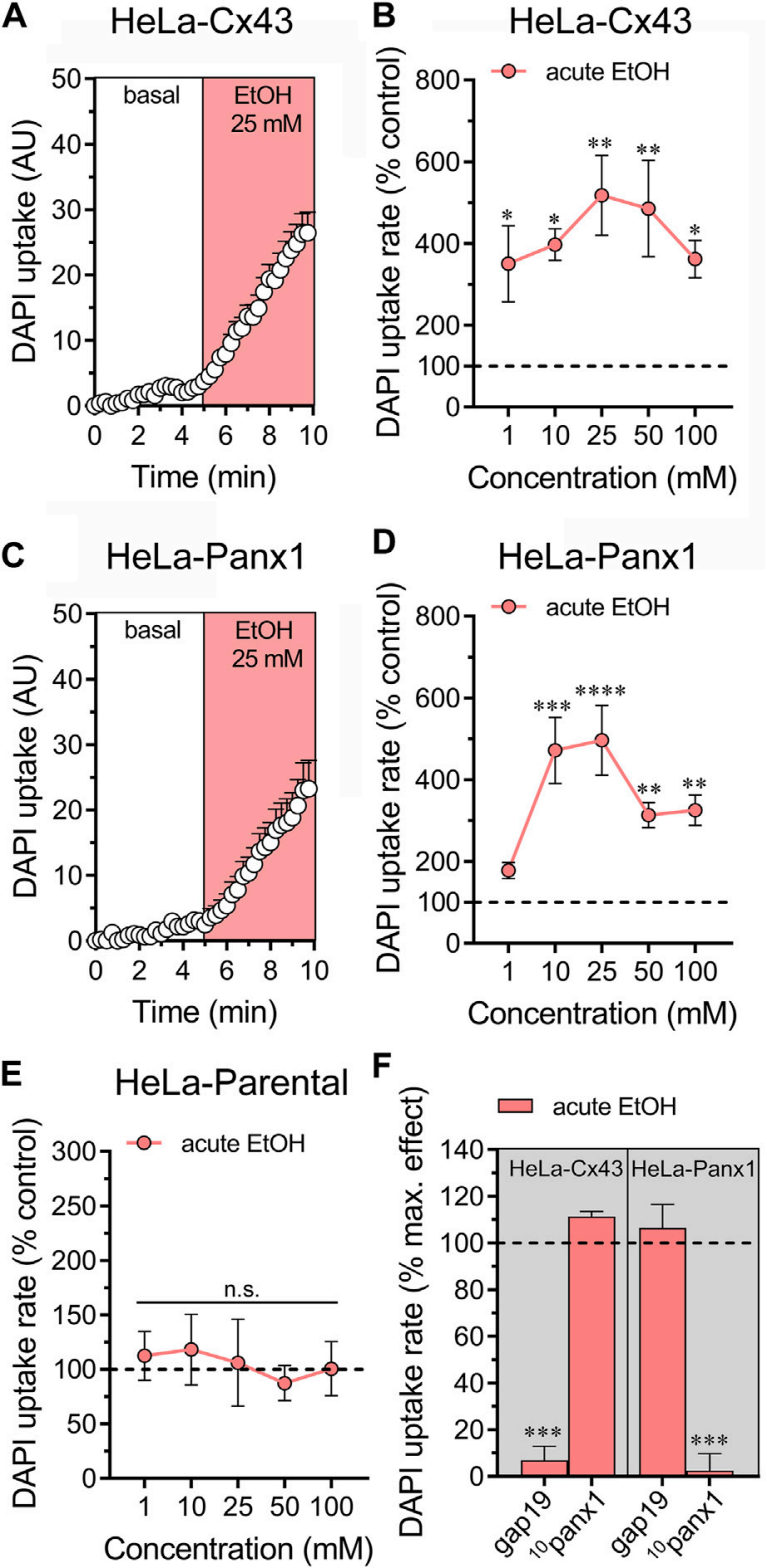
Ethanol acutely augments the activity of hemichannels in HeLa cells transfected with Cx43 or Panx1. **(A)** Time-lapse measurements of DAPI uptake by HeLa-Cx43^EGFP^ cells under basal conditions and then acutely exposed to 25 mM ethanol. **(B)** Averaged DAPI uptake rate normalized with the control condition (dashed line) by HeLa-Cx43^EGFP^ cells acutely treated with different concentrations of ethanol (salmon red circles). **(C)** Time-lapse measurements of DAPI uptake by HeLa-Panx1^EGFP^ cells under basal conditions and then exposed to 25 mM ethanol. **(D)** Averaged DAPI uptake rate normalized with the control condition (dashed line) by HeLa-Panx1^EGFP^ cells acutely treated with different concentrations of ethanol (salmon red circles). **(E)** Averaged DAPI uptake rate normalized with the control condition (dashed line) by HeLa parental cells acutely treated with different concentrations of ethanol (salmon red circles). **(F)** Averaged DAPI uptake rate normalized to the maximun effect evoked by 25 mM ethanol (dashed line) by HeLa-Cx43^EGFP^ or HeLa-Panx1^EGFP^ cells pretreated with the following blockers: 50 µM gap19 or 50 µM ^10^panx1. ***p < 0.0001, effect of pharmacological agents compared to ethanol treatment (two-tailed Student’s unpaired *t*-test). Data were obtained from at least three independent experiments with three or more repeats each one (≥15 cells analyzed for each repeat).

We previously showed that treatment for 24 h with ethanol increases the activity of Cx43 and Panx1 hemichannels expressed in HeLa cells (Gómez et al., 2024). To explore whether acute ethanol-induced hemichannel activity observed in astrocytes can be reproduced in an exogenous expression system, we transfected HeLa cells with Cx43 or Panx1 tagged with EGFP. These transfected cells express hemichannels on their cell surface, enabling them to uptake and release small molecules, including dyes commonly used to assess hemichannel activity, such as DAPI (Salas et al., 2015; Harcha et al., 2019). Acute ethanol treatment rapidly led to a six-fold increase in DAPI uptake in HeLa-Cx43^EGFP^ cells (Figures 2A,B). All ethanol concentrations tested (1–100 mM) significantly increased DAPI uptake, showing a bell-shaped pattern with 25 mM producing the maximum effect (Figure 2B). Similarly, HeLa-Panx1^EGFP^ cells exposed to acute ethanol displayed a similar bell-shaped increase in DAPI uptake, peaking at 25 mM, but significant effects were observed at concentrations equal to or greater than 10 mM (Figures 2C,D). Importantly, following stimulation with various ethanol concentrations, HeLa-parental cells (non-transfected) showed no alterations in DAPI uptake, indicating that acute ethanol specifically enhances the activity of Cx43 and Panx1 hemichannels in HeLa cells (Figure 2E). In line with these findings, 50 µM gap19 or 50 µM ^10^panx1 effectively blocked the acute ethanol-induced increase in DAPI uptake in HeLa-Cx43^EGFP^ and HeLa-Panx1^EGFP^ cells, respectively (Figure 2F).

### 3.2 Extracellular Ca^2+^ influx and Ca^2+^ release from intracellular stores contribute to the acute ethanol-induced activation of Cx43 and Panx1 hemichannels in cultured astrocytes

**FIGURE 3:**
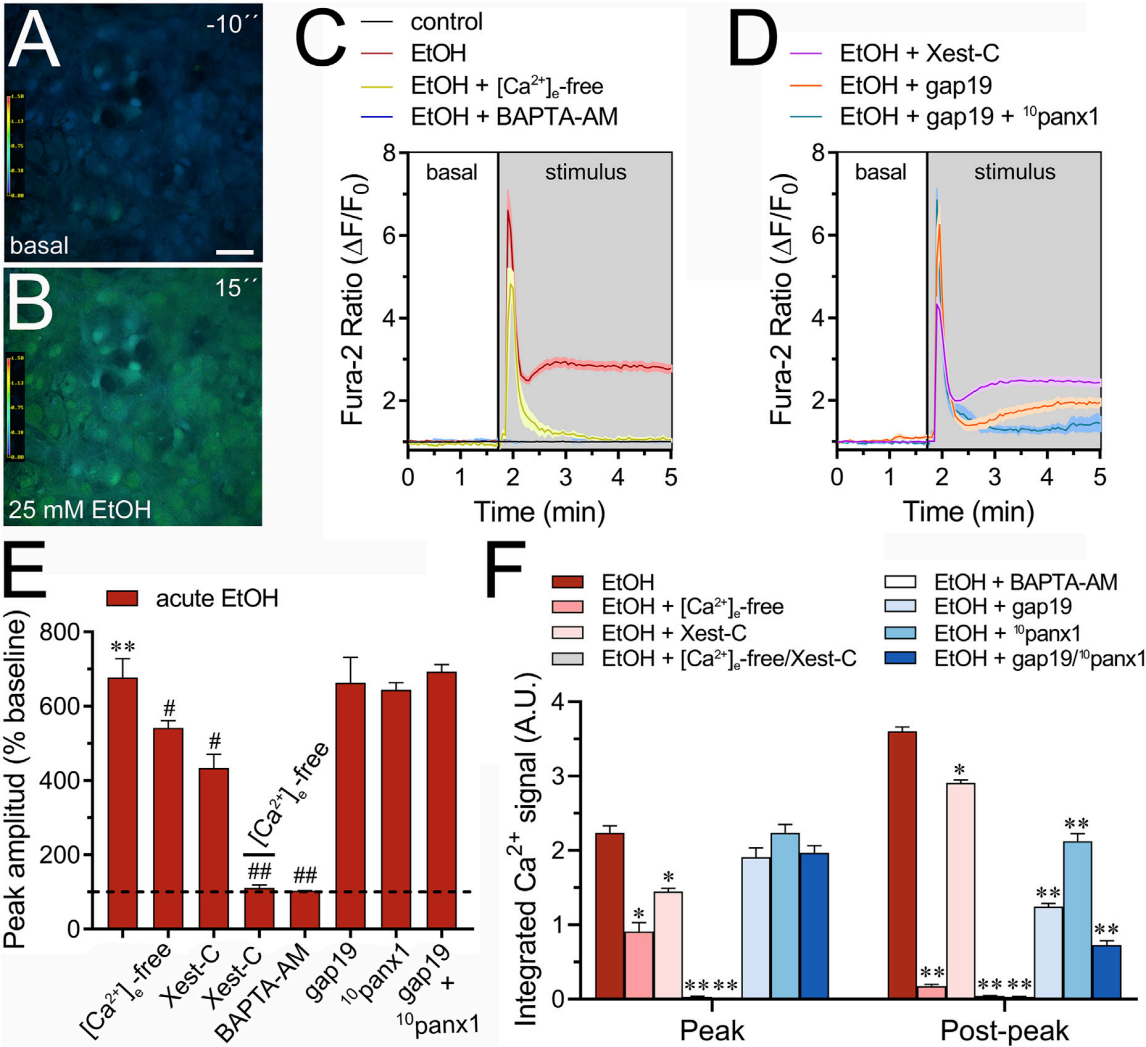
Cx43 and Panx1 hemichannels contribute to the acute ethanol-induced rise on [Ca^2+^]_i_ by cultured astrocytes. **(A–B)** Representative photomicrographs of Ca^2+^ signal (340/380 nm ratio) by astrocytes for 10 s before **(A)** and after stimulation with 25 mM ethanol for 15 s **(B)**. **(C–D)** Representative plots of relative changes in Ca^2+^ signal over time by astrocytes under control conditions (black line) or after the acute treatment with 25 mM ethanol (red line) alone or in combination with the following agents or conditions: [Ca^2+^]_i_-free bath solution (yellow line), 10 µM BAPTA-AM (purple line), 5 µM Xest-C (magenta line), 50 µM gap19 (orange line) and 50 µM gap19 + 50 µM ^10^panx1 (light blue line). **(E)** Averaged data of ethanol-induced peak amplitude by astrocytes normalized to basal Fura-2a.m. ratio. In addition, the effect of the following agents or conditions are shown: [Ca^2+^]_i_-free bath solution, 5 µM Xest-C, [Ca^2+^]_i_-free bath solution + 5 µM Xest-C, 10 µM BAPTA-AM, 50 µM gap19, 50 µM ^10^panx1, or 50 µM gap19 + 50 µM ^10^panx1. **p < 0.0005, ethanol compared to basal condition; ^#^ *p* < 0.05, ^##^ *p* < 0.005; effect of pharmacological agents compared to ethanol treatment (one-way ANOVA followed by Tukey’s *post hoc* test). **(F)** Averaged data of the area under the curve during and after the ethanol-induced peak of Ca^2+^ signal by astrocytes. In addition, the effect of the following agents or conditions are shown: [Ca^2+^]_i_-free bath solution, 5 µM Xest-C, [Ca^2+^]_i_-free bath solution + 5 µM Xest-C, 10 µM BAPTA-AM, 50 µM gap19, 50 µM ^10^panx1, or 50 µM gap19 + 50 µM ^10^panx1. *p < 0.05, **p < 0.005; effect of pharmacological agents compared to ethanol treatment (one-way ANOVA followed by Tukey’s *post hoc* test). Data were obtained from at least three independent experiments with three or more repeats each one (≥25 cells analyzed for each repeat). Calibration bar: 150 μm.

Moderate increases (>500 nM) in [Ca^2+^]_i_ trigger the opening of Cx43 hemichannels (De Bock et al., 2012), a response similarly observed with Panx1 hemichannels (Locovei et al., 2006; López et al., 2021). Furthermore, [Ca^2+^]_i_ dynamics regulate astroglial activation, function, and the release of gliotransmitters through various pathways (Volterra et al., 2014), including those associated with the opening of Cx43 hemichannels (Meunier et al., 2017). Given that acute ethanol exposure is known to rapidly elevate [Ca^2+^]_i_ in astrocytes (Allansson et al., 2001; Kim et al., 2022), we aim to investigate the molecular mechanisms underlying this phenomenon and whether hemichannels play a role in it. As depicted by time-lapse measurements of Fura-2 ratio (340/380), acute treatment with 25 mM ethanol elicited a rapid, intense, and transient increase in the Ca^2+^ signal, peaking at approximately 680% compared to basal levels (Figures 3A–C). This initial response was succeeded by a secondary or “post-peak” prolonged increase that gradually reached a plateau over the recording time (Figure 3C). To delve deeper into this response, we depleted the bath solution of Ca^2+^ or inhibited IP_3_-evoked Ca^2+^ release from intracellular Ca^2+^ stores using xestospongin C (Xest-C). Both the absence of extracellular Ca^2+^ or Xest-C (5 µM) attenuated the acute ethanol-induced increase in the amplitude and area under the curve of the [Ca^2+^]_i_ peak (Figures 3C–F). Notably, the secondary and persistent Ca^2+^ response triggered by acute ethanol was nearly completely blocked in the absence of extracellular Ca^2+^, while Xest-C only caused a slight reduction (Figures 3C,D,F). Remarkably, the combined depletion of extracellular Ca^2+^ and treatment with Xest-C completely abolished both the acute ethanol-induced [Ca^2+^]_i_ peak and the subsequent secondary [Ca^2+^]_i_ response (Figures 3E,F). Similar inhibitory effects were observed with BAPTA-AM (10 µM), an [Ca^2+^]_i_ chelator (Figures 3C, E, F). Together, these findings suggest that the rapid [Ca^2+^]_i_ peak triggered by ethanol in astrocytes relies on both extracellular Ca^2+^ influx and IP_3_-evoked Ca^2+^ release from intracellular Ca^2+^ stores. In contrast, the sustained secondary [Ca^2+^]_i_ response primarily depends on extracellular Ca^2+^ rather than Ca^2+^ released from intracellular stores.

**FIGURE 4:**
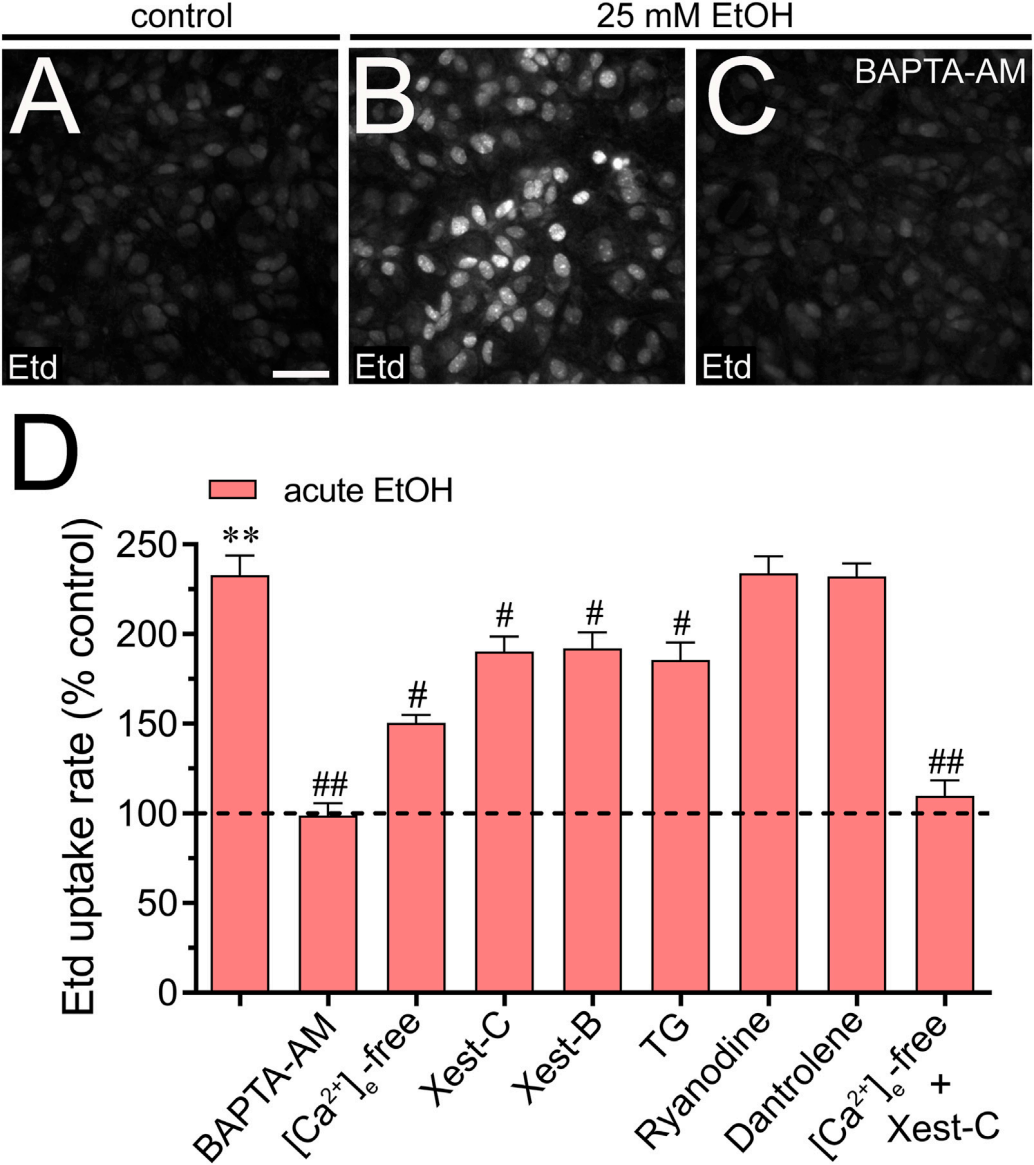
Extracellular Ca^2+^ influx and Ca^2+^ release from intracellular stores contribute to the acute ethanol-induced activation of Cx43 and Panx1 hemichannels in cultured astrocytes. **(A–C)** Representative fluorescence images depicting Etd uptake measurements (5 min of recording) in astrocytes under control conditions **(A)** or acutely treated with 25 mM ethanol **(B)** alone or plus 10 µM BAPTA-AM **(C)**. **(D)** Averaged Etd uptake rate normalized with control condition (dashed line) by astrocytes acutely treated with 25 mM ethanol alone or in combination with the following blockers or conditions: 10 µM BAPTA-AM, [Ca^2+^]_i_-free bath solution, 5 µM Xest-C, 5 µM Xest-B, 2 µM thapsigargin (TG), 100 µM ryanodine, 50 µM dantrolene or [Ca^2+^]_i_-free bath solution + 5 µM Xest-C. **p < 0.0005, ethanol compared to control; ^#^ *p* < 0.05, ^##^ *p* < 0.005; effect of pharmacological agents compared to ethanol treatment (one-way ANOVA followed by Tukey’s *post hoc* test). Data were obtained from at least three independent experiments with three or more repeats each one (≥25 cells analyzed for each repeat). Calibration bar = 150 μm.

We investigated the potential involvement of Cx43 and Panx1 hemichannels in the acute ethanol-induced rise in [Ca^2+^]_i_ signal using gap19 or ^10^panx1, respectively. Interestingly, neither gap19 (50 µM) nor ^10^panx1 (50 µM) affected the acute [Ca^2+^]_i_ peak elicited by ethanol (Figures 3D–F). However, the combined action of both inhibitors completely abolished the sustained secondary [Ca^2+^]_i_ response triggered by acute ethanol (Figures 3D–F). These findings, along with the inhibitory effect of the extracellular Ca^2+^-free solution, suggest that Cx43 and Panx1 hemichannels contribute to the persistent secondary extracellular Ca^2+^ influx triggered by acute ethanol. With this in mind, we then scrutinized whether the acute rise in [Ca^2+^]_i_ evoked by ethanol might be driving the heightened hemichannel activity caused by this substance. Both BAPTA-AM (10 µM) or the combined depletion of extracellular Ca^2+^ and treatment with Xest-C (5 µM) totally suppressed the acute ethanol-induced Etd uptake in astrocytes (Figures 4A–D). Partial inhibitory effects were observed upon depleting intracellular Ca^2+^ stores with thapsigargin (2 µM) or blocking IP_3_-evoked Ca^2+^ release from intracellular Ca^2+^ stores with Xest-C (5 µM) or xestospongin-B (5 μM, another IP_3_ receptor inhibitor) (Figure 4D). More pronounced blocking effects were observed upon depleting extracellular Ca^2+^ from the bath solution (Figure 4D). Interestingly, inhibiting ryanodine receptors (RyRs) with 100 µM ryanodine (a general RyR blocker) or 50 µM dantrolene (a specific RyR type-1 inhibitor) did not diminish the acute ethanol-mediated increase in Etd uptake by astrocytes (Figure 4D). Similarly, the presence of high extracellular K^+^ (50 mM) failed in modulate the acute ethanol-mediated increase in Etd uptake by astrocytes (Supplementary Figure S1E). Collectively, these findings emphasize that both extracellular Ca^2+^ influx and IP_3_-evoked Ca^2+^ release from intracellular Ca^2+^ stores cause the activation of hemichannels upon acute ethanol stimulation.

### 3.3 Acute ethanol-induced activation of Cx43 and Panx1 hemichannels leads to [Ca^2+^]_i_-dependent gliotransmitter release by astrocytes

**FIGURE 5:**
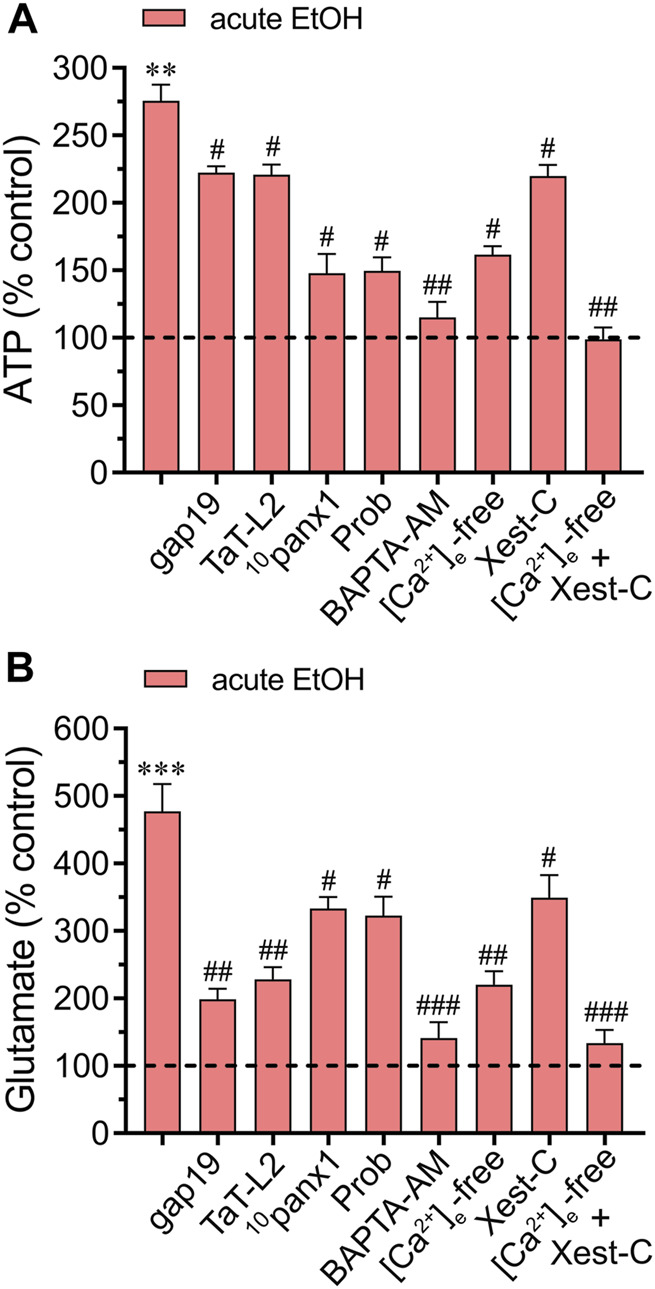
Acute ethanol exposure increases the release of gliotransmitters via the opening of Cx43 and Panx1 hemichannels. Averaged data of ATP **(A)** or glutamate **(B)** normalized to control conditions (dashed line) by astrocytes acutely treated with 25 mM ethanol alone or in combination with the following agents or conditions: 50 µM gap19, 50 µM TaT-L2, 50 µM ^10^panx1, 500 µM Probenecid (Prob), 10 µM BAPTA-AM, [Ca^2+^]_i_-free bath solution, 5 µM Xest-C or [Ca^2+^]_i_-free bath solution + 5 µM Xest-C. **p < 0.0005, ***p < 0.0001, ethanol compared to control; ^#^ *p* < 0.05, ^##^ *p* < 0.005, ^###^ *p* < 0.001; effect of pharmacological agents compared to ethanol treatment (one-way ANOVA followed by Tukey’s *post hoc* test). Data were obtained from at least three independent experiments with three or more repeats each one.

There is mounting evidence suggesting a rapid surge in extracellular ATP and glutamate levels during adverse brain conditions such as trauma (Globus et al., 1995; Davalos et al., 2005), hypoxia/ischemia (Nishizawa, 2001; Melani et al., 2005), or epilepsy-related seizures (Wieraszko and Seyfried, 1989; Albrecht and Zielinska, 2017). The prolonged elevation of ATP in brain dysfunction suggests controlled mechanisms of ATP release linked to the activation of danger signaling rather than mere leakage. Likewise, the excitotoxic release of glutamate is a characteristic hallmark implicated in mediating neuronal death due to exacerbated activation of excitatory amino acid receptors. Building upon prior research indicating that acute ethanol exposure boosts the release of ATP and glutamate by astrocytes (Salazar et al., 2008; Kim et al., 2021), we investigated this phenomenon within our system and explored the potential involvement of hemichannels. Examination of conditioned media (CM) obtained from astrocytes exposed to ethanol for 10 min showed elevated extracellular concentrations of ATP and glutamate compared to control conditions (Figures 5A,B). Significantly, ^10^panx1 or probenecid notably suppressed the acute ethanol-induced release of ATP (Figure 5A). Conversely, inhibition of Cx43 hemichannels (using gap19 or TaT-L2) elicited only a minor counteractive effect (Figure 5A). Interestingly, a different impact of hemichannels was noted in the release of glutamate compared to ATP levels when analyzing the CM. Certainly, we found that gap19 or TaT-L2 significantly dampened the acute ethanol-induced release of glutamate (Figure 5B). Additionally, inhibiting Panx1 hemichannels was effective, though to a lesser degree compared to blocking Cx43 hemichannels (Figure 5B). The next step was to figure out whether the acute ethanol-induced release of ATP and glutamate by astrocytes depended on [Ca^2+^]_i_. As occurred with the hemichannel activation elicited by acute ethanol exposure, both BAPTA-AM or the combined depletion of extracellular Ca^2+^ and treatment with Xest-C (5 µM) totally suppressed the acute ethanol-induced release of ATP and glutamate by astrocytes (Figures 5A,B). The [Ca^2+^]_i_ dependency response mentioned above was associated with extracellular Ca^2+^ influx rather than Ca^2+^ release from intracellular stores. This was evidenced by the fact that the inhibitory effect of Xest-C on gliotransmitter release was weaker compared to the depletion of extracellular Ca^2+^ (Figures 5A,B). Altogether, these findings suggest that acute ethanol exposure causes the release of gliotransmitters by astrocytes by a mechanism that involves the [Ca^2+^]_i_-dependent activation of Cx43 and Panx1 hemichannels.

**FIGURE 6:**
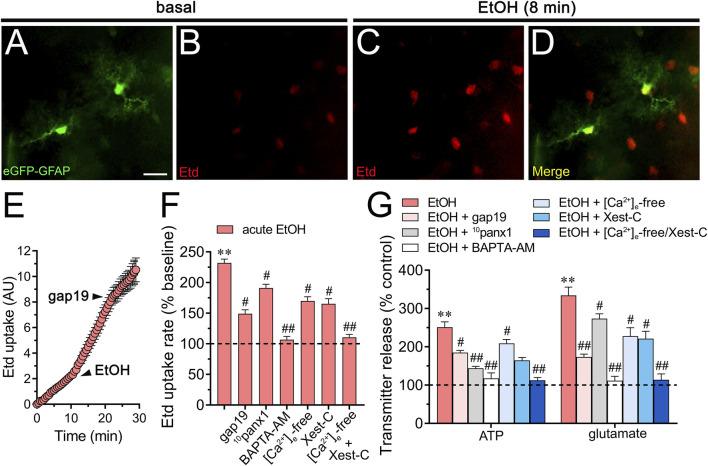
Acute ethanol exposure increases the release of ATP and glutamate in brain slices by a mechanism involving the activation of Cx43 and Panx1 hemichannels in a [Ca^2+^]_i_-dependent manner. **(A–D)** Representative fluorescence micrographs of Etd uptake (red) in hippocampal eGFP-GFAP astrocytes (green) from acute brain slices under basal conditions **(A, B)** and after the acute treatment with 25 mM ethanol for 8 min **(C, D)**. **(E)** Time-lapse measurements of Etd uptake by eGFP positive astrocytes under basal conditions, after the acute treatment with 25 mM ethanol and upon the acute treatment with 50 µM gap19. **(F)** Averaged Etd uptake rate normalized with control condition (dashed line) by eGFP positive astrocytes acutely treated with 25 mM ethanol alone or in combination with the following blockers or conditions: 50 µM gap19, 50 µM ^10^panx1,10 µM BAPTA-AM, [Ca^2+^]_i_-free bath solution, 5 µM Xest-C, or [Ca^2+^]_i_-free bath solution + 5 µM Xest-C. **p < 0.0005, ethanol compared to control; ^#^ *p* < 0.05, ^##^ *p* < 0.005; effect of pharmacological agents compared to ethanol treatment (one-way ANOVA followed by Tukey’s *post hoc* test). Data were obtained from at least three independent experiments with three or more repeats each one (≥5 cells analyzed for each repeat). **(G)** Averaged data of ATP (left) or glutamate (right) normalized to control conditions (dashed line) by astrocytes acutely treated with 25 mM ethanol alone or in combination with the following agents or conditions: 50 µM gap19, 50 µM ^10^panx1, 10 µM BAPTA-AM, [Ca^2+^]_i_-free bath solution, 5 µM Xest-C, or [Ca^2+^]_i_-free bath solution + 5 µM Xest-C. **p < 0.0005, ethanol compared to control; ^#^ *p* < 0.05, ^##^ *p* < 0.005; effect of pharmacological agents compared to ethanol treatment (one-way ANOVA followed by Tukey’s *post hoc* test). Data were obtained from at least three independent experiments with three or more repeats each one. Calibration bar = 60 μm.

To investigate the effects of acute ethanol on astrocytes within a more integrative setting, we investigated whether this substance could change hemichannel function in hippocampal astrocytes from the stratum radiatum in acute brain slices. Accordingly, we conducted time-lapse recordings of Etd uptake in eGFP-GFAP-positive astrocytes upon acute exposure to 25 mM ethanol. Hippocampal astrocytes exhibited a 2.3-fold rise in Etd uptake following acute ethanol treatment (Figures 6A–F). This increase was accompanied by elevated release of ATP and glutamate, as determined by luciferin/luciferase and colorimetric analysis, respectively, of CM from brain slices stimulated with 25 mM ethanol (Figures 6F,G). Both Etd uptake and transmitter release induced by acute ethanol were significantly reduced by gap19 (50 µM) or ^10^panx1 (50 µM) (Figures 6F,G). In line with findings from cultures, inhibiting Cx43 hemichannels proved more effective than blocking Panx1 hemichannels in reducing the release of glutamate induced by acute ethanol, whereas the opposite trend was observed for ATP release (Figure 6G). Likewise, both BAPTA-AM and the combined depletion of extracellular Ca^2+^ with Xest-C (5 µM) completely suppressed the acute ethanol-induced rise in Etd uptake and release of ATP and glutamate in brain slices (Figures 6F,G). In summary, these observations suggest that acute ethanol exposure induces the rapid activation of Cx43 and Panx1 hemichannels by hippocampal astrocytes in brain slices, resulting in the further release of ATP and glutamate in a [Ca^2+^]_i_-dependent manner.

## 4 Discussion

Here, we provide the first evidence that acute ethanol exposure triggers the rapid activation of Cx43 and Panx1 hemichannels in astrocytes. This heightened activity stems from a rapid rise in [Ca^2+^]_i_ linked to extracellular Ca^2+^ influx and IP_3_-evoked Ca^2+^ release from intracellular Ca^2+^ stores. Relevantly, the acute ethanol-induced activation of hemichannels contributes to a persistent secondary increase in [Ca^2+^]_i._ Furthermore, the [Ca^2+^]_i_-dependent activation of astroglial hemichannels elicited by ethanol leads to the notable release of ATP and glutamate in cultures and brain slices. Indeed, through the assessment of Etd uptake, we demonstrated that acute ethanol rapidly increases the activity of Cx43 and Panx1 hemichannels in a bell-shaped concentration-dependent manner. Treatment with gap19 and TaT-L2, broadly established mimetic peptides known to antagonize Cx43 hemichannel opening (Iyyathurai et al., 2013), significantly counteracted the acute ethanol-induced Etd uptake. Similar inhibitory effects were observed with the blockade of Panx1 hemichannels using ^10^panx1 or probenecid (Pelegrin and Surprenant, 2006; Silverman et al., 2008), highlighting the substantial contributions of both Cx43 and Panx1 hemichannels to rapid ethanol-induced Etd uptake. These observations were further confirmed using inactive and/or scramble forms of these peptides. Consistent with this, acute ethanol-induced activation of hemichannels was also observed in HeLa cells expressing exogenous Cx43^EGFP^ or Panx1^EGFP^, but not in parental HeLa cells devoid of both hemichannels. This indicates that the rapid activation of hemichannels evoked by ethanol depends entirely on the specific expression of Cx43 or Panx1 in an additive manner.

Previously, we reported that cultured astrocytes stimulated with ethanol for hours to days exhibit an enhanced activation of Cx43 and Panx1 hemichannels (Gómez et al., 2024). However, the temporal range employed in that study did not permit us to establish whether ethanol acutely modulates the function of astroglial hemichannels. Our current data are consistent with the notion that ethanol acutely boosts the activity of hemichannels with significant repercussions for [Ca^2+^]_i_ signaling and the release of relevant paracrine molecules by astrocytes. The concentration of ethanol (25 mM) that elicited the highest activation of astrocytic hemichannels in both cell cultures and brain slice preparations corresponds with clinically relevant blood alcohol levels (BALs) associated with binge drinking (White, 2003). The expression of both Cx26 and Cx30 has been reported in astrocytes (Nagy et al., 2003). While Cx30 can form hemichannels (Nielsen et al., 2017; Xu and Nicholson, 2023), functional Cx30 hemichannels in astrocytes have only been described in one report (Ghezali et al., 2020). Similarly, Cx26 forms functional hemichannels (Ripps et al., 2004; González et al., 2006), but to our knowledge, evidence demonstrating the Cx26 hemichannel activity in astrocytes is lacking. Further studies are needed to determine if the acute stimulatory effects of ethanol also occur in other hemichannel-forming proteins, such as Cx26 and Cx30.

How does acute ethanol stimulation cause the activation of Cx43 and Panx1 hemichannels in astrocytes? Early hypotheses suggested that ethanol’s lipid solubility primarily affects cell membranes, with secondary effects on cellular proteins (Samson and Harris, 1992). However, contemporary understanding reveals that ethanol interacts with ethanol-sensitive membrane proteins by binding to hydrophobic pockets, leading to conformational shifts or alterations in kinetics that affect protein function (Diamond and Gordon, 1997; Fadda and Rossetti, 1998). Changes in [Ca^2+^]_i_ homeostasis induced by ethanol have been identified as significant contributors to its effects on synaptic function and plasticity (Catlin et al., 1999; Walter and Messing, 1999). Here, we observed that acute ethanol-induced activation of hemichannels relies on both extracellular Ca^2+^ influx and IP_3_-evoked Ca^2+^ release from intracellular Ca^2+^ stores. These findings are coherent with previous studies showing that a moderate rise in [Ca^2+^]_i_ induces the activation of Cx43 and Panx1 hemichannels (Locovei et al., 2006; De Bock et al., 2012; López et al., 2021). Crucially, the phosphorylation of Panx1 amino acid residue S394 by Ca^2+^/calmodulin-dependent protein kinase II (CaMKII) is essential for the [Ca^2+^]_i_-dependent activation of Panx1 hemichannels (López et al., 2021). A similar mechanism has been proposed to occur during the [Ca^2+^]_i_-mediated activation of connexin hemichannels (Hu et al., 2020). The latter is relevant because CaMKII activation in response to ethanol occurs rapidly (<60 s) and robustly following the rapid increase of [Ca^2+^]_i_ (Garic et al., 2011). Future studies are needed to elucidate whether the acute ethanol-induced activation of Cx43 and Panx1 hemichannels occurs via the stimulation of CaMKII in astrocytes.

At this moment, the mechanisms underlying the acute ethanol-induced rise in [Ca^2+^]_i_ remain unclear. However, since only the combined depletion of extracellular Ca^2+^ and treatment with Xest-C completely blocked the acute ethanol-induced [Ca^2+^]_i_ peak, this phenomenon likely relies on extracellular Ca^2+^ influx and IP_3_-evoked Ca^2+^ release from intracellular Ca^2+^ stores. More importantly, our data suggest that the rapid [Ca^2+^]_i_ peak is followed by a persistent secondary influx of extracellular Ca^2+^ through Cx43 and Panx1 hemichannels. This aligns with prior studies indicating that hemichannels are permeable to Ca^2+^ and/or their activation leads to the indirect increase of [Ca^2+^]_i_ via activation of plasma membrane receptors and/or receptor-gated Ca^2+^ store (Schalper et al., 2010; Fiori et al., 2012; Lee et al., 2020; Yang et al., 2020). The rise in [Ca^2+^]_i_ regulate the function astrocytes (Volterra et al., 2014), including the release of gliotransmitters through several pathways (Goenaga et al., 2023). In this study, we showed that acute ethanol stimulation triggers the rapid release of ATP and glutamate through the activation of Cx43 and Panx1 hemichannels in a [Ca^2+^]_i_-dependent manner. Interestingly, we noted that acute ethanol induces a differential release of ATP or glutamate depending on the hemichannel-forming protein (Cx43 or Panx1) in both culture and brain slice preparations. Specifically, inhibiting Panx1 hemichannels was more effective in reducing ATP release, whereas the opposite trend was observed for glutamate release when compared to inhibiting Cx43 hemichannels. We reported a similar gliotransmitter release behavior in cultured astrocytes treated for hours to days with ethanol (Gómez et al., 2024). The latter could be explained by the fact that, although both channels allow Etd passage, they may play different roles in releasing ATP and glutamate in our setting. While this might seem puzzling, recent studies suggest that hemichannels are not indiscriminate non-selective pores; rather, they selectively permeate certain molecules in an isoform-specific manner (Hansen et al., 2014a; Hansen et al., 2014b; Nielsen et al., 2017). Therefore, the uptake of fluorescent dyes may not precisely correlate with permeation to ions or small biologically relevant molecules, a characteristic that could even vary under pathological conditions (Gaete and Contreras, 2020; Sáez et al., 2020).

Acute ethanol intoxication poses a persistent hazard to society, leading to traffic accidents, injuries, and violence. It is widely documented that acute ethanol exposure has varied effects on neuronal activity across different brain regions, including the striatum, hippocampus, cerebellum, amygdala, substantia nigra, and ventral tegmental area (Abrahao et al., 2017). This impairs decision-making, judgement, and cognitive control, and can cause amnestic episodes, commonly referred to as memory “blackouts,” associated with binge drinking (White, 2003; George et al., 2005). Memory blackouts can have profound personal and societal consequences, particularly among adolescents and young adults, and may predict further cognitive difficulties with persistent alcohol use (White, 2003; Parada et al., 2011). This and other adverse brain effects can persist in the alcohol hangover period when BAL levels have dropped to zero (Silvestre de Ferron et al., 2015; Gunn et al., 2018; Contreras et al., 2019). The natural release of gliotransmitters from astroglial hemichannels regulates fundamental aspects of synaptic transmission, plasticity, memory, learning, and behavior (Chever et al., 2014; Meunier et al., 2017; Cheung et al., 2022; Linsambarth et al., 2022; Vasile et al., 2022). Therefore, it is plausible to hypothesize that the rapid release of gliotransmitters induced by ethanol through these channels could reconfigure synaptic networks and circuits, leading to unexpected effects on memory and behavior. Our findings offer fresh perspectives on the potential mechanisms behind acute alcohol-induced brain abnormalities and propose targeting Cx43 and Panx1 hemichannels in astrocytes as a promising avenue for treating such conditions.

## Data Availability

The original contributions presented in the study are included in the article/Supplementary Material, further inquiries can be directed to the corresponding authors.
