# Supplementary figures and images for: Acute activation of hemichannels by ethanol leads to Ca2+-dependent gliotransmitter release in astrocytes
Source: Front Cell Dev Biol. 2024 Jun 21;12:1422978. doi: 10.3389/fcell.2024.1422978 (PMC11224458; doi:10.3389/fcell.2024.1422978)

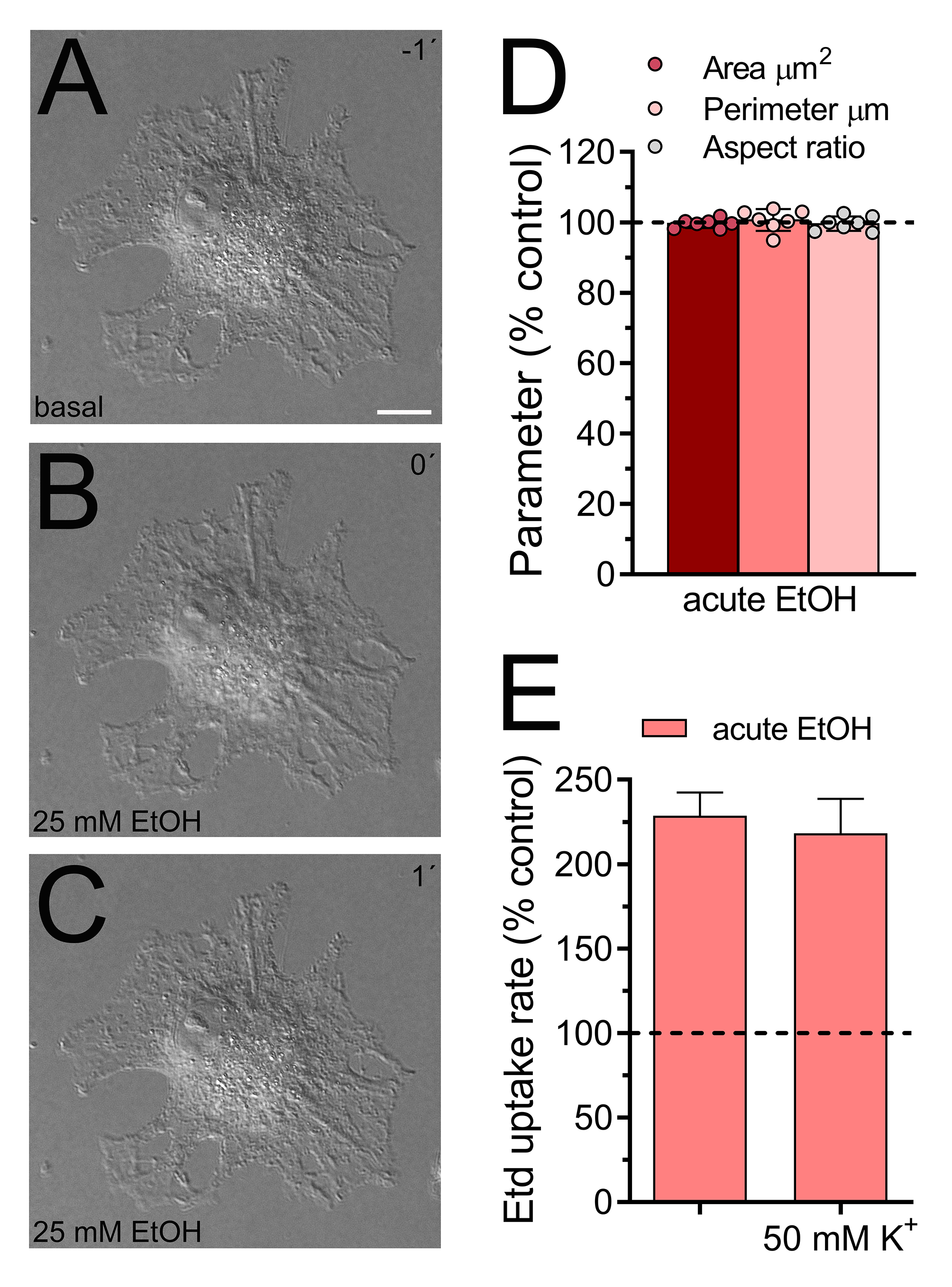

Supplement: Supplementary file 1 [file Image1.TIF]
